# P-glycoprotein (ABCB1) and Oxidative Stress: Focus on Alzheimer's Disease

**DOI:** 10.1155/2017/7905486

**Published:** 2017-11-26

**Authors:** Giulia Sita, Patrizia Hrelia, Andrea Tarozzi, Fabiana Morroni

**Affiliations:** ^1^Department of Pharmacy and Biotechnology, Alma Mater Studiorum-University of Bologna, Via Irnerio 48, 40126 Bologna, Italy; ^2^Department for Life Quality Studies, Alma Mater Studiorum-University of Bologna, Corso d'Augusto, 237, 47900 Rimini, Italy

## Abstract

ATP-binding cassette (ABC) transporters, in particular P-glycoprotein (encoded by ABCB1), are important and selective elements of the blood-brain barrier (BBB), and they actively contribute to brain homeostasis. Changes in ABCB1 expression and/or function at the BBB may not only alter the expression and function of other molecules at the BBB but also affect brain environment. Over the last decade, a number of reports have shown that ABCB1 actively mediates the transport of beta amyloid (A*β*) peptide. This finding has opened up an entirely new line of research in the field of Alzheimer's disease (AD). Indeed, despite intense research efforts, AD remains an unsolved pathology and effective therapies are still unavailable. Here, we review the crucial role of ABCB1 in the A*β* transport and how oxidative stress may interfere with this process. A detailed understanding of ABCB1 regulation can provide the basis for improved neuroprotection in AD and also enhanced therapeutic drug delivery to the brain.

## 1. Introduction

The rise in life expectancy, with the relative aging of the population, involves serious demographic, ethical, social, economic, and medical problems. In particular, the incidence of neurodegenerative diseases has increased considerably by ten or fifteen years to this part. Common to all of the neurodegenerative disorders is the irreversible degeneration of distinct subsets of neurons, the accumulation of aggregated peptides, and the imbalance of cellular oxidative state.

Alzheimer's disease (AD) is the most common cause of dementia and one of the most important causes of morbidity and mortality among the aging population. The appearance of beta amyloid (A*β*) plaques in the extracellular compartment of the brain parenchyma is a hallmark of AD, and biochemical and genetic findings highlight the crucial role of the A*β* peptide in the pathogenesis of AD [1]. In addition to the recognized pathological signs of senile plaques and neurofibrillary tangles, the presence of extensive oxidative stress (OS) is a contributing factor in the progression of AD. The accumulation of free radical damage and alterations in the activities of antioxidant enzymes are also present in AD patients [2]. However, the exact mechanisms by which the redox balance is altered and the sources of free radicals in the AD brain are still unknown. It has been demonstrated that A*β* is capable of promoting the formation of ROS through a mechanism that involves the PI3K/Akt/GSK3 and MAPK/ERK1/2 pathways [3] and that OS may increase A*β* production and aggregation as well facilitate tau phosphorylation, forming a vicious cycle that promotes the progression of AD [4].

The question to ask is why the A*β* peptide accumulates in the brain. There are two possible explanations: (i) the overproduction of A*β* in the brain and (ii) the reduced clearance of A*β* from the brain [5, 6]. Only familial AD (5% of cases) is due to the overproduction of A*β* because of mutations in the amyloid precursor protein (APP) gene or in the APP processing enzymes [7, 8], while the greater part (95%) of the so-called sporadic AD cases are probably caused by dysfunctions in A*β* aggregation, degradation, and removal [5, 9]. It has been proposed that the underlying cause of A*β* accumulation in AD is a reduced clearance of A*β* from the brain via the blood-brain barrier (BBB) [10, 11].

ATP-binding cassette (ABC) transporters are multidomain integral membrane proteins that use the energy of ATP hydrolysis to translocate solutes across cellular membranes in all mammalian species [12]. In the last decade, a number of reports have shown that members of the ABC superfamily of membrane proteins, in particular P-glycoprotein (encoded by ABCB1), actively mediate the transport of A*β* [13]. Cirrito et al. [14] demonstrated that the deficiency of ABCB1 at the BBB increased A*β* deposition in an AD mouse model, suggesting that A*β* is transported out of the brain or periarterial interstitial fluid through this transport system.

Although many studies on ABCB1 and AD are present in the literature, the link between OS and ABC membrane transport systems, during aging and in OS-related diseases, as AD, is still unclear, thus providing an urgent need for a deeper understanding of mechanisms through which such processes and diseases develop. In this review, we discuss the possible role of ABCB1 and OS in AD and consider how a fuller understanding of these aspects might promote the development of more effective treatment strategies.

## 2. Blood-Brain Barrier and Oxidative Stress

In the human body, the brain represents the most sensitive organ to OS, not only because its own proper function requires the precise control of the extracellular environment but also because of the huge demand for nutrients by the brain itself. Indeed, the oxygen requirements of the brain tissue accounts for approximately 20% of the total human oxygen consumption [15]. The BBB is an essential biochemical and physical barrier that separates the central nervous system (CNS) from the bloodstream and plays a fundamental role in the balance of the brain microenvironment. Indeed, it maintains the ion balance and the low gradient of excitatory neurotransmitters, regulates the transport of specific nutrients, and limits the entry of toxic substances both endogenous and exogenous [13]. This is essential for a reliable synaptic transmission and an effective neuroregulation activity. In this view, it promotes the longevity of the SNC and prevents premature death and cellular neurodegeneration [16].

This barrier is mainly formed by a monolayer of tightly junctioned endothelial cells. Anyway, this is not enough to form a functionally BBB per se, which requires the presence of interaction with adjacent glial cells as well as neurons, pericytes, and collagen extracellular matrix [17, 18]. This intricate relationship between both vascular and neuronal cells is called implied neurovascular unit (NVU). The NVU avoids the entry of compounds from the circulating blood to the brain via paracellular or transcellular diffusion. For this reason, the brain homeostasis is maintained through specific transporters or passive diffusion mechanisms [19]. In fact, oxygen, carbon dioxide, glucose, nucleosides, vitamins, and part of liposoluble drugs can reach the SNC, but it has been reported that the BBB is responsible for blocking the delivery of more than 98% of drugs [20–22].

Several neurodegenerative diseases are characterized by increased inflammation. Indeed, neuroinflammation exacerbates the pathology by generating inflammatory mediators, as well as by activating microglia, and by the production of reactive oxygen species (ROS). All together, these events contribute to spread OS [23, 24]. The resulting condition is that the BBB tight junctions are wrecked, causing a consistent variation in brain microenvironment [25].

Microglia, as the first and primary active immune defense in the CNS, express multiple subfamilies of ABC transporters and are particularly sensitive to brain injury or disease and switch their morphology and phenotype to an “activated” state in response to brain insults [26, 27].

Among ROS, the superoxide anion, a by-product of physiological processes, contribute to BBB endothelial dysfunction [28–30]. In normal conditions, superoxide dismutase (SOD) enzyme regulates biological activity of superoxide, but under oxidative conditions, the anion is produced at high levels that overcome the metabolic capacity of SOD. BBB damage can be intensified by conjugation of superoxide and nitric oxide (NO) to form peroxynitrite, a cytotoxic and proinflammatory molecule. Peroxynitrite causes significant injury to microvessels through lipid peroxidation, consumption of endogenous antioxidants, and induction of mitochondrial failure [31, 32]. Overall, OS contributes to disruption of endothelial cell-cell interactions and to BBB injury by promoting redistribution or downregulation of critical tight junction proteins such as claudin-5, occludin, zonula occludens-1, and junctional adhesion molecule-1 [33–36].

In particular, the importance of brain-to-blood transport of brain-derived metabolites across the BBB has gained increasing attention as a potential mechanism in the pathogenesis of neurodegenerative disorders.

## 3. ABC Transporters

The cells forming the NVU achieve the control of brain homeostasis and microenvironment by the expression of complex active transport systems, such as ion channels, pumps, receptors, and transporters on the luminal or abluminal side of the BBB [37]. Despite the presence of BBB, small molecules and macromolecules could be transported into the brain to maintain its homeostasis. There are three main classes of BBB transporters: carrier-mediated transporters (CMT), active efflux transporters (AET), and receptor-mediated transporter (RMT). The CMT and AET systems are mainly responsible for the transport of small molecules, while the RMT systems are reserved for large molecules and involve endocytic transport. AET transporters include ABC family members, which form one of the largest of all protein families and are central to many important biomedical phenomena, including resistance of cancers and pathogenic microbes to drugs [38]. In fact, ABC transport system regulates drug bioavailability, metabolism, and distribution in cells and in the extracellular matrix, limiting substrate cellular influx and retention [39–42]. ABC proteins share a common molecular structure composed by nucleotide-binding domains, which conserved peptide motifs, and transmembrane domains, usually composed of six transmembrane helices [43]. In the BBB, ABC transporters are localized on the blood-facing plasma membrane where they allow unidirectional transport from the cytoplasm to the extracellular space. This localization can be considered as a strategy to protect the brain against the numerous lipophilic xenobiotics which, because of their chemical structure, should rapidly diffuse across endothelial cell membranes [42, 44–46].

The human ABC superfamily includes 48 multidrug transporters, belonging to one of the seven ABC A to ABC G subfamilies. These subfamilies include the ABCA member 1 (ABCA1) that acts as cholesterol efflux regulatory protein (CERP) and extrudes phospholipids from cell membranes to Apolipoprotein E (ApoE); the ABCB subfamily member 1 (ABCB1), which mediates multidrug resistance (MDR); the ABCC subfamily of multidrug resistance-associated proteins (MRP); and the ABCG2 subfamily of the breast cancer resistance protein (BRCP) [40]. Depending on their subfamily, these transporters act either as “protectors” or as “vehicle” for bioactive molecules produced by cells [45].

The first transporter to be identified and studied was the ABCB1, a phosphorylated glycoprotein of 170 kDa usually localized on the luminal side of the brain capillary endothelial cells. In humans, it is coded from the mdr1 gene, of which there are more than 50 polymorphisms at the level of single nucleotide. Because of its highly polymorphic nature, this gene is responsible for a strong variability in drug absorption and tolerance [47]. Among these polymorphisms, the rs1128503 is a nucleotide change in exon 12 (C1236T) that does not alter the glycine at position 412, while the triallelic rs2032582 polymorphism in exon 21 (G2677 T/A) leads to an alanine to threonine or serine amino acid substitution (Ala893Thr/Ser). Finally, the rs1045642 synonymous polymorphism in exon 26 (C3435T) does not affect the leucine at position 1145 [48]. To the best of our knowledge, the main products of this gene are two, ABCB1 and ABCB2, but only the first one confers multidrug resistance [49]. This is due to the fact that ABCB2 is commonly expressed by hepatocytes at the canalicular side for the secretion of phosphatidylcholine into the bile fluid [50].

Because of the primary sequence of ABCB1, that contains the characteristic short ATP-binding motifs and in between an additional conserved sequence characteristic of ABC superfamily, ABCB1 has been classified as ABC transporters. ABCB1 is localized in both the luminal and abluminal membranes of brain capillary endothelial cells to carry out its role as “brain sentinel” [51–54]. It is still not clear if the expression of ABCB1 is only restricted to those brain blood vessels that are part of the BBB, or if it is equally expressed in fenestrated capillaries of the circumventricular organs (area postrema in the brainstem, the subfornical organ, the median eminence, the pineal body, the vascular organ of the lamina terminalis, choroid plexus, and neurohypophysis) [55, 56].

Several studies have demonstrated that rat astrocytes and microglial cultures can express multiple membrane transporters including ABCB1, but in lower levels when compared to brain endothelial cells culture [57–59]. Differently, many studies demonstrated the expression of ABCB1 at microglia, astrocytes, and pericytes adjacent to endothelial cells in normal primate brains [60–62]. These authors reported that ABCB1 was distributed along the nuclear envelope, in the caveolae, in cytoplasmic vesicles, and in Golgi complex and rough endoplasmic reticulum. On the other hand, numerous studies did not detect ABCB1 in neuronal or glial cells, suggesting that its expression in these cells may depend by pathological CNS conditions from seizures to tumors [63–67].

Besides its protective function, ABCB1 has also been implicated in resistance to apoptosis. The programmed cell death contributes to tissue remodeling and to elimination of damaged cells, and through the stimulation of this pathway, ABCB1 may affect the regenerative process in lesioned tissue. Several mechanisms that could explain this event have been described. First, ABCB1 might block the caspase-3 activation by inhibiting caspase-8 induction that is normally conducted by Fas. As shown by Ruefli et al. [68], this event needs ATP binding or hydrolysis, because mutations in the ATP-binding regions abolished ABCB1-mediated Fas resistance. Second, ABCB1 may affect the apoptosis induced by ceramide. Indeed, when ceramide is not converted into the nontoxic glucosylceramide, it mediates cell death. ABCB1 promotes the translocation of the nontoxic derivate from the cytosolic to the luminal face of the Golgi and in this way influences directly ceramide metabolism and, indirectly, causes apoptosis resistance [69, 70].

In the beginning, this drug efflux pump was discovered by oncologists as responsible for chemotherapy resistance, but besides this ability, ABCB1 confers resistance to numerous drugs, including immunosuppressive drugs, HIV protease inhibitors, and antibiotics [71–73].

ABCB1 function could be counteracted with competitive inhibitors, such as verapamil and cyclosporine A, or directly by blocking its function, as with elacridar (GF120918) [74–78]. It has been shown that a clinically relevant oral dose of oxytetracycline is able to saturate ABCB1 and, subsequently, to increase the absorption of other drugs [79]. Several side effects may be associated with the inhibition of this transporter activity. A dramatic example is congestive heart failure caused by a combined therapy of verapamil and doxorubicin to inhibit ABCB1 [80]. The use of efflux transporters inhibitors, when applied coincidently for general treatment or to enhance CNS uptake of drugs, are better suited to acute therapies where the aim is to reach short maximal concentrations, as happens in the treatment of brain tumors, than for chronic administration where long-term inhibition will interfere with the normal brain homeostasis. In this context, it is also interesting to consider a recent review by Kalvass et al. [81], in which authors discussed the low probability of modulating transporters at the human BBB by currently marketed drugs. The central conclusion of their work is that, while increased CNS distribution of efflux transport substrates has been commonly observed in animal models and when dosed with nonmarketed inhibitors (e.g., tariquidar) in humans, the overall clinical evidence indicates that drug interactions at the human BBB due to efflux transporter inhibition by marketed drugs are low in magnitude (≤2-fold increase in brain : plasma ratio). Moreover, serious adverse CNS safety events arising from these interactions have not been observed.

Looking at the cerebral proteopathies, a long inhibitory treatment may lead to raised intracerebral concentrations of a large spectrum of neurotoxic substances that enter the brain or are directly being produced within the brain. These changes of the transport kinetics and the accumulation of neurotoxins species should take in the study of the pathogenesis of neurodegenerative diseases, where dysfunction of ABCB1 has been associated with Parkinson's disease (PD), progressive supranuclear palsy (PSP), multisystem atrophy (MSA), and with depressive disorders [82–85].

Whereas ABCB1 has long dominated the “stage” of transporter-related drug resistance in cancer, other ABC transporters became clinically relevant in the 1990s. In particular, the ABCC family includes 13 related ABC transporters that are able to transport structurally different lipophilic anions. The most intriguing feature of ABCCs is that they provide a transport facility for compounds (drugs, xenobiotics, or physiological substrates) conjugated with glutathione (GSH), glucuronide, or sulfate. For several tissues and cell types, the release of glutathione disulfide (GSSG) has been reported during OS and has been proposed to be an endogenous mechanism of cellular defense [86, 87]. Among ABCC transporter subfamily, ABCC1–4 are known to be responsible for GSH and GSSG transport [88–90]. Indeed, studies in primary cultures of rat astrocytes showed that MK571, a known inhibitor of ABCC, blocks the transport of GSH [91]. Despite the fundamental role of ABCC family in the detoxifying cellular system, in this context, our attention is focalized on the role of ABCB1 and OS in AD.

## 4. ABCB1 and Oxidative Stress in Alzheimer's Disease

Apart from the fact that ABCB1 remains one of the major cause of chemotherapy resistance in cancer, this transport protein play an important role as export pump of endogenous compounds and exogenous toxic agents in a variety of cells and tissues. Because of its high levels of expression at the BBB, ABCB1 is a powerful gatekeeper to the brain. We can affirm that ABC transporters in general, and ABCB1 in particular, have evolved to counteract OS, indeed toxic compounds generated are eliminated by ABC transporters after they are detoxified by conjugation to GSH, glucuronide, and sulfate. Interestingly, following organ damage or disease, changes in the expression levels of ABC transporters have been observed, probably to compensate the increased load of OS products or to compensate for the loss of efflux pumps in damaged tissues. Alteration of ABCB1 participated in many CNS disorders, such as upregulation in epilepsy [92], neural inflammation [93], and stroke [94]. DeMars et al. [95] demonstrated that middle cerebral artery occlusion increased ABCB1 in the liver in conjunction with increased ABCB1 in the brain. Certainly, understanding mechanisms and signals that modulate ABCB1 expression and activity at the BBB could result in new therapeutic targets for CNS disorders. Many sensors of the cellular and extracellular environment are capable of changing ABC transporter expression at the BBB, in particular oxidative and inflammatory stress, diet, pharmacotherapy, and toxicant exposure [96].

**Figure 1:**
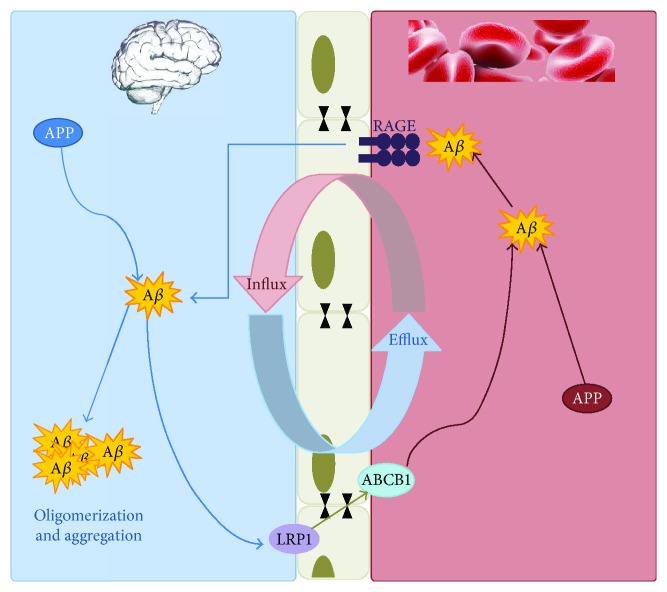
Proposed components of the A*β* efflux and influx across the blood-brain barrier. The two main A*β* efflux transporters include the low-density lipoprotein receptor-related protein 1 (LRP-1) and ABCB1, and the receptor for advanced glycation end-products (RAGE) is the main A*β* influx transporter.

It is known that ROS have been implicated in the regulation of ABCB1. However, there is still substantial controversy about the association between ABCB1 expression and OS. Conditions that generate ROS have been shown to increase ABCB1 expression in the liver [97] and kidney [98]. Conversely, other studies have demonstrated decreased ABCB1 due to increased ROS levels in tumor spheroids [99]. The actual amount of ROS seems to be essential in determining what cellular effects are initiated. Thus, in conditions of OS, ROS may function as an endothelial signal transduction intermediate promoting cell survival, increasing ABCB1 expression. However, ROS may also lead to increased lipid peroxidation, which is implicated in BBB disintegration, with a consequent decrease in ABCB1 expression and activity. OS is a cofactor in nearly every CNS disorders and there is substantial evidence that the accumulation of ROS can acutely disrupt the BBB. Felix and Barrand demonstrated the effect of OS on the expression of ABCB1 at BBB endothelium [100]. They exposed primary cultured rat brain endothelial cells to hydrogen peroxide, causing a concentration-dependent increase in expression and activity of ABCB1. A subsequent study by the same group proposed the involvement of several signaling effectors including ERK1/2, Akt, and JNK, which in turn activated nuclear factor-*κ*B (NF-*κ*B). Consistent with these results, Hong et al. demonstrated that ABCB1 expression was upregulated under conditions of chronic OS-induced GSH depletion in rat brain capillary endothelial cells [101]. These effects were reversed by the ROS scavenger, N-acetylcysteine, suggesting that depletion of GSH leads to elevated ROS, which induces ABCB1 expression. ROS can play a crucial role in signal transduction [102] through various transcriptional factors, such as NF-*κ*B and nuclear factor E2-related factor-2 (Nrf2). In turn, these transcription factors can regulate the expression of ABC transporters. Nrf2 is a transcription factor that regulates the expression of proteins that protect against OS. Nrf2 normally resides in the cytoplasm bound to Kelch-like ECH-associated protein 1 (Keap1). Upon oxidant/electrophile binding on Keap1, Nrf2 is released and translocates to the nucleus where it binds to antioxidant response elements, increasing expression of key players in the antioxidant response, including genes that code for proteins that produce GSH, reduce ROS, and metabolize xenobiotics. Importantly, administering Nrf2 ligands is neuroprotective in animal models of neurological disorders, such as cerebral ischemia, subarachnoid hemorrhage, spinal cord injury, PD, and AD [103–107]. Wang et al. demonstrated that Nrf2 activation with the isothiocyanate sulforaphane (SFN) in vivo or in vitro increases expression and transport activity of ABCB1 at the BBB. Dosing rats with SFN increased ABCB1 expression in the brain capillaries and decreased by 50% brain accumulation of the ABCB1 substrate, verapamil. No such effects were seen in the brain capillaries from Nrf2-null mice, indicating Nrf2 dependence [108]. Oxidative damage is a relative early event in the pathogenesis of AD. Several findings support the hypothesis that A*β* interferes with oxidative phosphorylation, which results in OS and apoptosis in brain cells [109, 110]. According to the amyloid cascade hypothesis, AD results from the accumulation of A*β* in the brain [1]. The neurovascular hypothesis of Zlokovic et al. states that a critical pathological event driving A*β* accumulation in the brain is the reduced clearance of A*β* from the brain across the BBB [111]. Over the last 10 years, a new AD research field emerged with a focus on ABC transporters at the BBB and in other cells of the CNS. Lam et al. [112] were the first to demonstrate that the potent and efficient efflux transporter ABCB1 is able to transport A*β*. Pharmacological blockade of ABCB1 rapidly decreased extracellular levels of A*β* secretion. They were able to directly measure transport of A*β* peptides across the plasma membranes of ABCB1-enriched vesicles and showed that this phenomenon was both ATP- and ABCB1-dependent. The transport of A*β* at the BBB is bidirectional. As shown in Figure 1, the two main A*β* efflux transporters include the low-density lipoprotein receptor-related protein 1 (LRP-1) and ABCB1, and the receptor for advanced glycation end-products (RAGE) is the main A*β* influx transporter [14, 113].

As demonstrated by Hartz et al. [114], extracellular A*β* first comes into contact with LRP-1 on the abluminal side of the brain endothelial cell. This is followed by transport of A*β* into the vascular lumen by ABCB1, or by a ABCB1-independent pathway. Entry of circulating A*β* into the brain is mediated by RAGE, but can also be restricted by ABCB1 [115]. The mode of interaction between A*β* and ABCB1 is not clear yet. Previous studies raised two possibilities: (i) ABCB1 could mediate A*β* transport directly or (ii) ABCB1 could interact with A*β* but does not transport it. In this case, ABCB1 may anchor A*β* on the plasma membrane and inhibit uptake into the endothelial cells [116]. In pathological conditions, as AD, it is possible to observe a reduction of ABCB1 at the BBB, which is probably associated with the accumulation of A*β* in the brain. As previously demonstrated by Loo and Clarke [117], the ubiquitin-proteasome pathway is responsible, at least in part, for the regulation of ABCB1 trafficking, localization, stability, and functions. In a recent work, Hartz et al. [118] focused on the critical mechanistic steps involved in the reduction of ABCB1 in AD. They investigated in the brain capillaries if A*β*_40_ triggers ABCB1 ubiquitination, internalization, and proteasome-dependent degradation leading to reduction of ABCB1 expression and activity. Indeed, experiments with microtubule and proteasomal inhibitors confirmed that ABCB1 was internalized and degraded by the proteasome. As the authors expected, A*β*_40_ activates the ubiquitin–proteasome system at the BBB, resulting in ABCB1 degradation and in a reduction of its expression and activity levels.

Moreover, it is possible to hypothesize that diminished ABCB1 expression due to increasing age, genetic, or environmental factors may lead to impaired A*β* clearance, followed by the accelerated accumulation of intracerebral A*β* and eventually the development of AD.

Cirrito et al. [14] provided the first evidence of a direct link between BBB, ABCB1, and A*β* brain deposition. Using *Mdrla/b^−/−^* double-knockout mice, the authors demonstrated that brain clearance of A*β* was significantly lower compared to that in control animals after intracerebroventricular injection of A*β*. Then, the authors dosed transgenic hAPP-overexpressing mice, a well-established AD model, with a selective ABCB1 inhibitor and measured A*β* brain concentrations by microdialysis. As expected, A*β* levels in the brain interstitial fluid were significantly increased compared to untreated hAPP control mice. Moreover, isolated brain capillaries from transgenic Tg2576 mice showed a 70% decrease in ABCB1 transport activity and a 60% decrease in ABCB1 protein expression compared to age-matched wild-type mice [114]. Consistent with these in vivo studies, a significant negative correlation exists between the densities of senile plaque and ABCB1 levels in the brain capillaries of patients with AD [119]. Interestingly, using ^11^C-verapamil and positron emission tomography imaging, a clinical study showed significant reduction in ABCB1 activity in AD patients compared to cognitively normal subjects [120]. Another study detected 25% lower ABCB1 protein expression levels in hippocampal blood vessels in postmortem brain samples from AD patients than in samples from age-matched nondemented patients [121]. Despite all these data supporting the involvement of decreased ABCB1 activity in A*β* accumulation in AD, little is known about the mechanisms that could initiate or sustain these transport deficiencies in disease progression. Some studies pointed at A*β* accumulation itself as a causative factor [122, 123]. Park et al. proved that A*β* mediated ABCB1 downregulation in murine brain endothelial cells by RAGE activation. These authors suggested that activation of RAGE by A*β* would enhance NF-*κ*B activity that decreases ABCB1 expression [124]. It is possible that in the early stages of the disease, accumulation of A*β* levels in the brain capillary plasma membrane could directly impair ABCB1 function, lead to A*β* accumulation, and reduce ABCB1 expression. Hartz et al. [125] suggested that A*β* contributes to the loss of BBB integrity that could be responsible for BBB dysfunction and cognitive decline and to the compromised integrity of both specific membrane transporters and proteins of the tight junction complex. Altered BBB homeostasis not only causes neuronal damage but also compromises A*β* clearance at the NVU, resulting in a vicious cycle between A*β* accumulation and BBB dysfunction during AD progression [126]. Although BBB disruption is often detected in AD patients, it is not clear whether it is a specific feature of AD. In this regard, further studies should be conceived to define how BBB function is altered before AD onset and during disease progression. A deeper understanding of how BBB dysfunction is a cause or consequence in AD pathogenesis could allow the development of new therapeutic strategies targeting BBB for this neurodegenerative disease.

In our opinion, also OS and neuroinflammation may play a pivotal role in these transport deficiencies. The AD brain is in a chronic proinflammatory state, and indeed, A*β* causes inflammation in the brain through Toll-like receptor and complement activation [127], and elevated levels of proinflammatory cytokines and acute phase proteins are localized around A*β* plaques [128]. It was reported that ABCB1 downmodulates the function of dendritic cells, which are considered to be crucial regulators of specific inflammatory processes through the secretion of proinflammatory cytokine, resulting in an impaired immune response. This finding suggests a new physiological role for ABCB1 as an immunomodulatory molecule and reveals a possible new target for immunotherapy [129].

Oligomeric A*β* can also generate OS by producing the lipid peroxidation product, 4-hydroxynonenal [130], and through activation of NADPH oxidase, the superoxide-producing enzyme, in microglia [131]. On the other hand, inflammation and/or OS can themselves cause A*β* accumulation in the brain, thus creating a vicious circle. Indeed, OS upregulates proteins involved in A*β* production, such as presenilin 1 [132].

Erickson et al. [133] speculated that downregulation of BBB efflux transporters, as ABCB1, in AD may represent a pathological consequence of prolonged vascular sequestration of A*β* as a result of sustained systemic oxidative and inflammatory state. This possibility is supported by Hartz et al. who showed in a transgenic model of AD that ABCB1 dysfunction at the BBB preceded symptoms of cognitive impairment [114]. It is possible that aging would likely sensitize an organism to inflammation and OS so that the threshold required for A*β* efflux impairment is lowered [134]. Thus, targeting intracellular signals that upregulate ABCB1 in the early stages of AD has the potential to increase A*β* clearance from the brain and reduce its accumulation.

## 5. What Therapeutic Perspectives?

Taking into account all these considerations, the concept that restoring ABCB1 at the BBB could be a valid therapeutic strategy to lower A*β* brain load, reduce cognitive decline, delay onset, and slow progression of AD has to be critically evaluated. The involvement of ABCB1 in the clearance of A*β* was demonstrated by Brukmann et al. [135]. The authors showed that the absence of ABCB1 results in a significant disturbance of A*β* removal in a transgenic murine model of AD (APP/PS1^+^/^−−^ABCB1), leading to an increased intraparenchymal cerebral amyloid angiopathy. We need to consider that if on the one hand, the BBB and ABCB1 are both neuroprotective; on the other hand, they are substantial obstacles to the delivery of drugs to the CNS. In this view, a recent work suggested that the simultaneous administration of verapamil, a known inhibitor of ABCB1 activity, and berberine, a promising natural anti-inflammatory and antioxidant compound, significantly potentiated their neuroprotective effect on behavioral alterations, OS, mitochondrial dysfunction, neuroinflammation, and histopathological modifications in a streptozocin-induced rat model of sporadic dementia [136].

Thus, increased ABCB1 expression enhances neuroprotection, but at the expense of drug delivery; on the contrary, reduced transporter activity decreases neuroprotection, but provides opportunity to increase drug delivery to the CNS [137]. This dual role of ABCB1 should be carefully considered in AD patients, because elderly patients often have a combination of several chronic diseases and need proper drug delivery.

For less than 10 years, the literature reported studies in AD research to demonstrate the neuroprotective activity of compounds acting on ABCB1. In 2009, Nishida et al. [138] crossed AD transgenic (APPsw) model mice with *α*-tocopherol transfer protein knock-out (Ttpa^−/−^) mice in which lipid peroxidation in the brain was significantly increased. The resulting double-mutant (Ttpa^−/−^APPsw) mice showed increased A*β* deposits in the brain, which was ameliorated with *α*-tocopherol supplementation. Interestingly, the A*β* generation in Ttpa^−/−^APPsw mouse brain was not increased, but the authors considered that accumulated A*β* in Ttpa^−/−^APPsw mouse brain was caused by these impaired A*β* clearance. A*β* aggregation was accelerated in these mice compared with wild-type, while LRP-1 and ABCB1 were upregulated in the small vascular fraction of AD mouse brains, probably to compensate their dysfunctions to transport increased toxic substrates in the brain caused by lipid peroxidation.

Moreover, in a transgenic mouse model of AD (human amyloid precursor protein- (hAPP-) overexpressing mice; Tg2576 strain), brain capillary ABCB1 expression and transport activity were substantially reduced compared with wild-type control mice, suggesting a mechanism by which A*β* accumulates in the brain in AD. Treatment of 12-week-old asymptomatic hAPP mice for 7 days with pregnenolone-16*α*-carbonitrile to activate the nuclear receptor pregnane X receptor (PXR) restored ABCB1 expression and transport activity in the brain capillaries and significantly reduced the brain A*β* levels compared with untreated control mice [114]. PXR is activated by a number of drugs and dietary constituents, and potent ligands for human PXR include the antibiotic rifampin and the St. John's wort (SJW) constituent hyperforin [139]. In this regard, a clinical trial showed that rifampin dosing lessened cognitive decline in patients with AD over the 12-month treatment period [140]. The mechanistic basis for this observation is not known, but rifampicin activation of PXR leading to induction of ABCB1 in BBB is a likely possibility.

Another compound that modulates ABCB1 activity is oleocanthal, a phenolic component of extravirgin olive oil. The authors provided in vitro and in vivo evidences for the potential of oleocanthal to enhance A*β* clearance from the brain via upregulation of ABCB1 and LRP1 at the BBB. In cultured mice brain endothelial cells, oleocanthal treatment increased ABCB1 and LRP1 expression and activity. Brain efflux index (BEI%) studies of ^125^I-A*β*_40_ showed that administration of oleocanthal extracted from extravirgin olive oil to C57BL/6 wild-type mice enhanced ^125^I-A*β*_40_ clearance from the brain and increased the BEI% from 62.0 ± 3.0% for control mice to 79.9 ± 1.6% for oleocanthal-treated mice [141]. In a further study, the same authors confirmed its effect in the hippocampal parenchyma and microvessels of TgSwDI mice, a transgenic model of AD [142].

In 2014, Brenn et al. [143] clarified the effect of SJW on the accumulation of A*β* and ABCB1 expression in the brain. The authors showed that long-term administration (60 and 120 days) of SJW extract (final hyperforin concentration 5%) leads to a significant reduction of soluble A*β*_1–42_ (representing mainly small oligomers and monomers) as well as A*β*_40_- and A*β*_42_-positive plaque number and size (representing mainly fibrillar and protofibrillar A*β*), while vascular ABCB1 expression was increased in the brains of double transgenic mice.

The expression of ABCB1 at the BBB is also regulated by the vitamin D receptor (VDR). In 2011, Chow et al. [144] showed that mice treated with the physiological ligand of VDR, the 1*α*,25-dihydroxytitamin D [1,25(OH)_2_D_3_] had lower accumulation of digoxin, an ABCB1 substrate. Similarly, one year later Durk et al. [145] demonstrated *in vitro* not only that rat brain endothelial cells (RBE4) and human (hCMEC/D3) cerebral microvessels endothelial cells incubated with 1,25(OH)_2_D_3_ showed an increase in ABCB1 expression but also that the treatment counteracted the brain accumulation of A*β*. These findings were confirmed by a second study conducted in 2014 by the same authors [146] with two transgenic mouse models of AD, one at a preplaque formation age (Tg2576) and the other at a plaque formation or already formed age (TgCRND8). In this study, Durk et al. showed that ABCB1 expression via VDR activation decreased soluble A*β* and reduced plaque formation in young TgCRND8 mice, improving also conditioned fear memory. However, the treatment of old TgCRND8 mice (after plaque formation), even if was able to decrease soluble A*β*, it did not reduce the plaque burden. The study underlined not only the role of ABCB1 in AD pathogenesis but also the importance of VDR on its regulation.

Another class of compounds identified as inducer for ABCB1 activity was suggested by Manda et al. [147], which demonstrated in LS-180 cells that fascalpsyn, a marine-derived bis-indole alkaloid, along with its 4,5-difluoro, induced a consistent fold increase in ABCB1 expression. Moreover, these compounds showed an inhibitory activity on acetylcholinestease (AChE), an enzyme strictly involved in the neuronal loss observed in AD patients.

After a study where the activity of rivastigmine to decrease A*β* accumulation was showed, in 2016, Mohamed et al. [148] continued their work by crossing the transgenic AD mouse model APPSWE with mdr1a/b knockout mice to assess rivastigmine activity on three different levels of ABCB1 expression (APP^+^/mdr1^+/+^, APP^+^/mdr1^+/−^, and APP^+^/mdr1^−/−^). The authors showed that the treatment with rivastigmine increased the expression of ABCB1 and LRP1 in isolated brain capillaries of APP^+^/mdr1^+/+^ and APP^+^/mdr1^+/−^. Interestingly, ABCB1 deletion caused a significant increase in parenchymal accumulation of A*β*_40_, but not A*β*_42_, when compared to APP/mdr1 wild-type mice. This inverse correlation between A*β*_40_ deposition and ABCB1 expression suggests the importance of ABCB1 to maintain A*β* brain homeostasis across the BBB.

The studies we have taken into consideration are summarized in Table 1 and are all very interesting and encouraging, but we believe that they need further investigation. Besides many neuroprotective effects of these compounds, the induction of ABCB1 thus might enhance A*β* clearance from the brain and thereby reduce the risk of developing AD. To test this hypothesis, further studies are warranted to investigate the effects of these compounds for example on animal behavior and memory. However, these studies indicated that the induction of ABCB1 is a promising therapeutic approach to the treatment and/or prevention of neurodegenerative diseases such as AD.

## 6. Conclusions

In summary, it is now clear that expression/activity of ABCB1 at the BBB is strictly related to A*β* clearance and eventually with AD progression. An understanding of transporter regulation is critical before we can determine to what extent signaling can be manipulated to improve not only drug delivery to the CNS but also to enhance neuroprotection. Although promising results in animal studies have been achieved, a better understanding into the signaling cascade of this transporter may result in a better understanding of AD etiology and in the development of novel therapeutic strategies.

## Figures and Tables

**Table 1a:** (a) *In vitro*

Treatment	Dose	Duration	Cell line	Species	Reference
Sulforaphane (SFN)	0.01–5 *μ*M	0–200 min	Brain capillaries	Nrf2^−/−^ mouse	[108]
				p53^−/−^ mouse	
Oleocanthal (Oleo)	0.5–50 *μ*M	72 h	bEnd3	Mouse	[141]
1*α*,25-Dihydroxyvitamin D3 (1,25(OH)_2_D3)	1–100 nM	4 h or 24 h	Brain capillaries	Rat	[145]
		1–3 days	RBE4	Rat	
			hCMEC/D3	Human	
Fascalpsyn	1–100 *μ*M	24–48 h	LS-180	Human	[147]
			hGF		

**Table 1b:** (b) *In vivo*

Treatment	Dose	Duration	Model	Species	Reference
SFN	1–10 mg/kg intraperitoneal (i.p.)	2 days	Sprague-Dawley	Rat	[108]
Verapamil	2–5 mg/kg i.p.	21 days	Streptozocin-induced sporadic dementia	Rat	[136]
Berberine	25–100 mg/kg per oral (p.o.)	21 days			
*α*-Tocopherol	36 mg/kg p.o.	Lifespan	Ttpa^−/−^ APPsw	Mouse	[138]
Pregnenolone-16*α*-carbonitrile	25 mg/kg i.p.	7 days	APPsw/Tg2576	Mouse	[114]
Oleo	10 mg/kg/12 h i.p.	2 weeks	C57BL/6	Mouse	[141]
Oleo	5 mg/kg/die i.p.	4 weeks	TgSwDI	Mouse	[142]
St. John's wort	1250 mg/kg/die p.o.	60–120 days	APP/PS^+/−^	Mouse	[143]
1,25(OH)_2_D3	2.5 *μ*g/kg/die i.p.	8 days	Fxr^−/−^	Mouse	[144]
1,25(OH)_2_D3	2.5 *μ*g/kg/48 h i.p.	8 days	Tg2576	Mouse	[146]
			TgCRND8		
Rivastigmine	0.3 mg/kg/day alzet pump	8 weeks	APP^+^/mdr1	Mouse	[148]

**Table 1c:** (c) Clinical trials

Treatment	Dose	Duration	Diagnosis	Reference
Rifampin	300 mg/die p.o.	12 months	AD	[140]
Doxycyclin	200 mg/die p.o.			
